# Supplementary material for: The Fungicidal Action of Micafungin is Independent on Both Oxidative Stress Generation and HOG Pathway Signaling in Candida albicans
Source: Microorganisms. 2020 Nov 26;8(12):1867. doi: 10.3390/microorganisms8121867 (PMC7768384; doi:10.3390/microorganisms8121867)
Supplement: Supplementary file 1 [file microorganisms-08-01867-s001.zip › microorganisms-1009710-supplementary.pptx]

## Slide 1
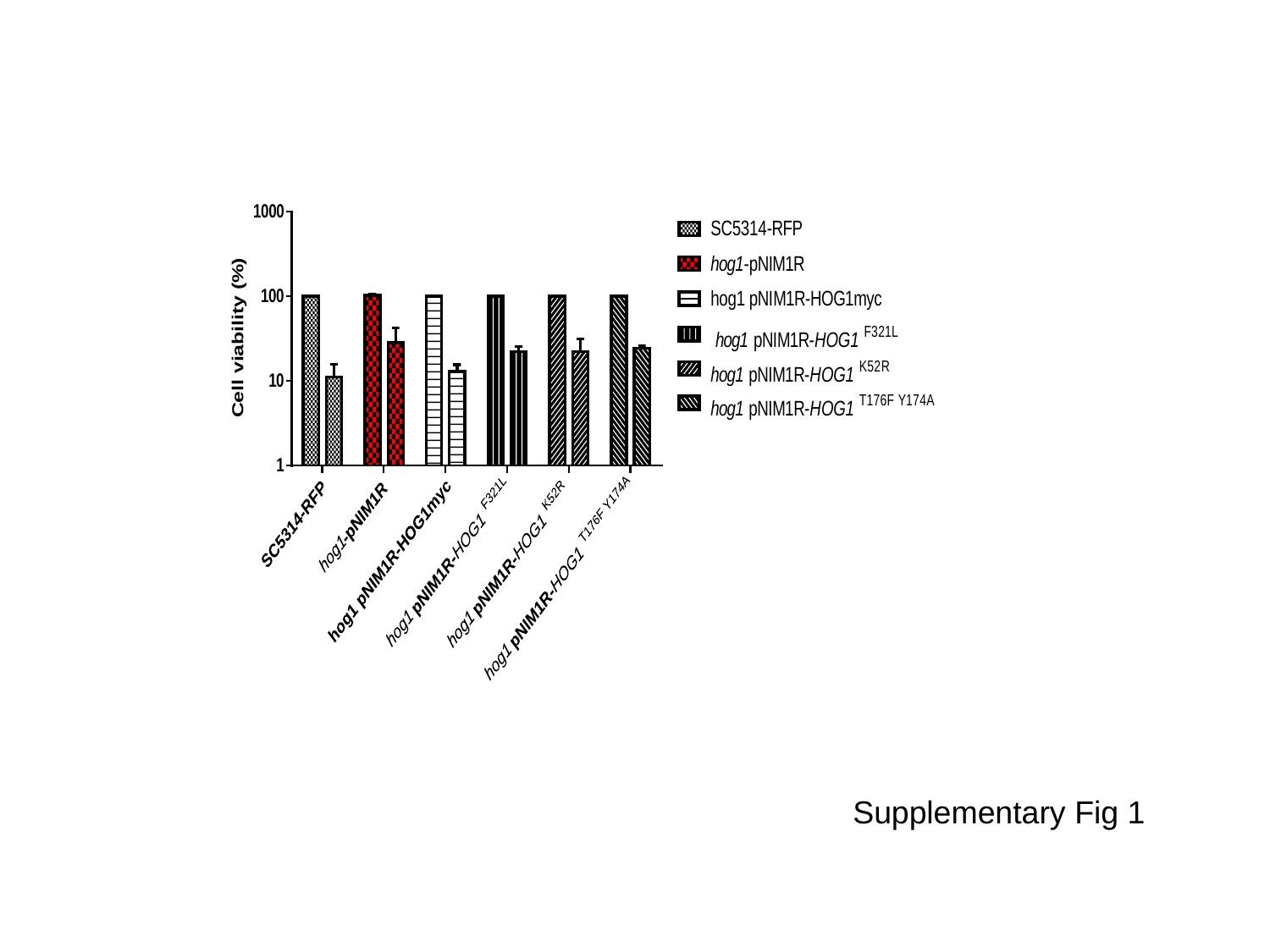

Supplementary Fig 1

## Slide 2
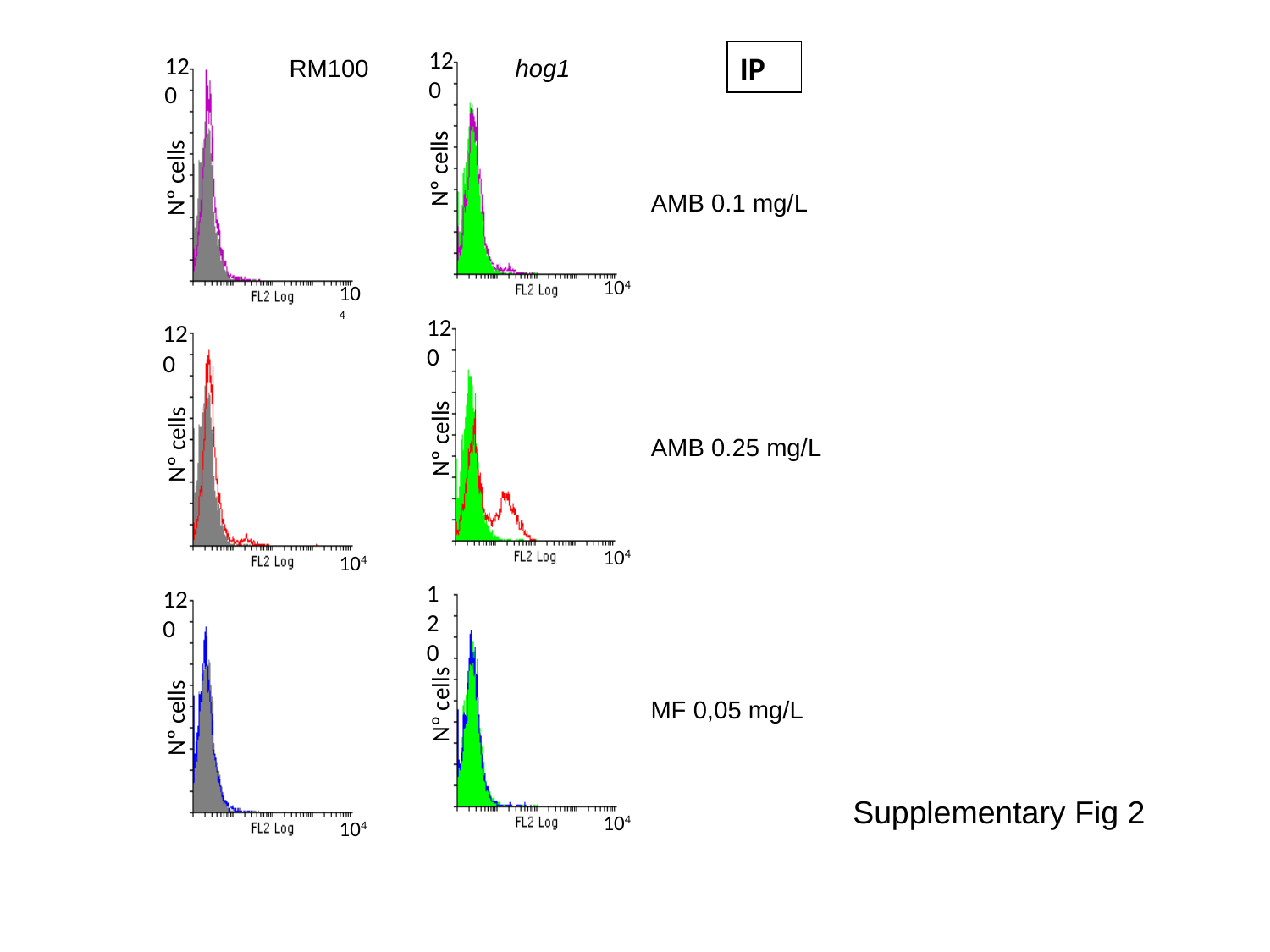

120
Nº cells
104
120
Nº cells
104
120
Nº cells
104
IP
120
Nº cells
104
120
Nº cells
104
120
Nº cells
104
RM100
hog1
AMB 0.1 mg/L
AMB 0.25 mg/L
MF 0,05 mg/L
Supplementary Fig 2
